# 3D genome organization links non-coding disease-associated variants to genes

**DOI:** 10.3389/fcell.2022.995388

**Published:** 2022-10-20

**Authors:** Gisela Orozco, Stefan Schoenfelder, Nicolas Walker, Stephan Eyre, Peter Fraser

**Affiliations:** ^1^ Centre for Genetics and Genomics Versus Arthritis, Division of Musculoskeletal and Dermatological Sciences, School of Biological Sciences, Faculty of Biology, Medicine and Health, The University of Manchester, Manchester Academic Health Science Centre, Manchester, United Kingdom; ^2^ NIHR Manchester Biomedical Research Centre, Manchester University Foundation Trust, Manchester, United Kingdom; ^3^ Enhanc3D Genomics Ltd., Cambridge, United Kingdom; ^4^ Epigenetics Programme, The Babraham Institute, Babraham Research Campus, CB22 3AT Cambridge, Cambridge, United Kingdom; ^5^ Department of Biological Science, Florida State University, Tallahassee, FL, United States

**Keywords:** 3D genomics, GWAS, gene expression, SNP, causality, promoter-capture Hi-C

## Abstract

Genome sequencing has revealed over 300 million genetic variations in human populations. Over 90% of variants are single nucleotide polymorphisms (SNPs), the remainder include short deletions or insertions, and small numbers of structural variants. Hundreds of thousands of these variants have been associated with specific phenotypic traits and diseases through genome wide association studies which link significant differences in variant frequencies with specific phenotypes among large groups of individuals. Only 5% of disease-associated SNPs are located in gene coding sequences, with the potential to disrupt gene expression or alter of the function of encoded proteins. The remaining 95% of disease-associated SNPs are located in non-coding DNA sequences which make up 98% of the genome. The role of non-coding, disease-associated SNPs, many of which are located at considerable distances from any gene, was at first a mystery until the discovery that gene promoters regularly interact with distal regulatory elements to control gene expression. Disease-associated SNPs are enriched at the millions of gene regulatory elements that are dispersed throughout the non-coding sequences of the genome, suggesting they function as gene regulation variants. Assigning specific regulatory elements to the genes they control is not straightforward since they can be millions of base pairs apart. In this review we describe how understanding 3D genome organization can identify specific interactions between gene promoters and distal regulatory elements and how 3D genomics can link disease-associated SNPs to their target genes. Understanding which gene or genes contribute to a specific disease is the first step in designing rational therapeutic interventions.

## 1 Introduction

### 1.1 The 3D genome regulates gene expression

#### 1.1.1 Gene expression depends on folding and proximity

**FIGURE 1:**
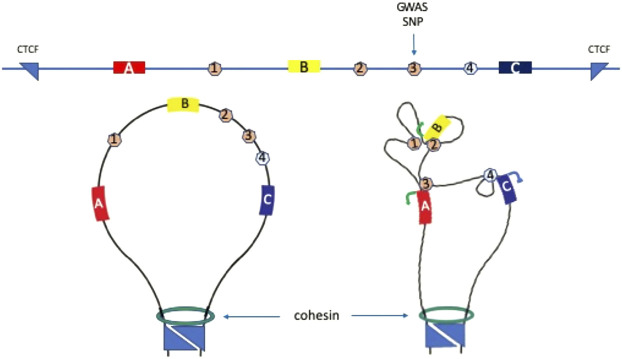
Gene regulatory elements and 3D genome conformation. Top line shows schematic of a hypothetical one megabase region of genome with three genes, **(A,B, and C)**; three enhancers 1, 2, and 3; one silencer element 4; and one genome wide association study (GWAS) single nucleotide polymorphism (SNP) (vertical arrow). The locus is flanked by convergent CTCF sites which interact and function as topologically associated domain (TAD) boundaries in cooperation with cohesin (bottom left panel). The GWAS SNP would normally be assigned to gene **(B)** or **(C)** due to proximity, however analysis of 3D genome data shows that SNP-bearing enhancer 3 contacts gene A promoter to activate transcription (bottom right panel). Enhancers 1 and 2 contact gene **(B)** to activate transcription. The silencer element 4 contacts gene **(C)** to silence transcription.

Cell type-specific gene expression depends upon promoters, enhancers, silencers, insulators and other gene regulatory elements that determine when genes are switched on, to what level they are expressed, and when they are switched off (Schoenfelder and Fraser, 2019). These regulatory sequences work in concert with trans-acting factors that bind to them to control the flow of genetic information from DNA to RNA to protein, thereby directing developmental, and differentiation cell fate decisions, as well as the maintenance of homeostasis and health. Understanding which regulatory sequences control which gene(s) requires an intricate knowledge of the 3D folding and arrangement of the genome in the cell nucleus, since all these elements carry out their functions through close proximity, direct interaction or contact. For example, enhancer sequences, which can activate or increase expression of a gene, can be located upstream, downstream or in the intron of a gene, and can operate over very large genomic distances to control the expression of a gene millions of base pairs away. Analysis of the sequences in the immediate spatial vicinity of a transcriptionally active gene in the nucleus revealed very close proximity of its distal enhancers, while the intervening sequences were located further away (Carter et al., 2002). This suggests formation of a loop, where regulatory activity of the enhancer is communicated to its target gene through direct interaction with its promoter (Figure 1) (Carter et al., 2002). “Engineered looping” between a gene promoter and distal enhancer sequence, by expression of factors that bind to each and can interact or dimerize, showed that transcription is indeed controlled by the enhancer-promoter loop, mediated by direct contact between factors bound to the regulatory sequences (Figure 1) (Deng et al., 2012; Morgan et al., 2017; Kim et al., 2019).

Enhancers have been mapped throughout the genome in a variety of cell types, using histone marks and cofactor binding. The human genome is thought to contain around one million enhancers (ENCODE project Consortum, 2012; Shen et al., 2012), greatly exceeding the number of protein-coding genes. Depending on the post-translational modifications of nucleosomal histone proteins in the immediate vicinity, enhancers can exist in several states. The neutral/intermediate state is marked by mono-methylation of lysine 4 on histone H3 (H3K4me1). The poised state is marked by H3K4me1 and trimethylation of lysine 27 on histone H3 (H3K27me3) and the active state is marked by H3K4me1 and acetylation of lysine 27 on histone H3 (H3K27ac) (Creyghton et al., 2010; Rada-Iglesias et al., 2011; Zentner et al., 2011). Silencer elements are less well-studied and, in some cases, may act in a manner similar to enhancers, by interacting directly with their target gene promoters (Figure 1). However, other mechanisms for silencers have also been observed or proposed (Segert et al., 2021). Insulators bind the CTCF factor and appear to block enhancer activation of a gene when located in the genomic sequence between the enhancer and the gene.

#### 1.2.1 Topologically associated domains; sub-chromosomal organization

CTCF insulator sequences are often found in clusters at the boundaries of large chromatin domains known as topologically associated domains (TADs). TADs average about 1 megabase (Mb) in size and vary little from cell type to cell type in structure, unlike enhancer-promoter loops which are cell type specific (Javierre et al., 2016). Both CTCF and its ring-shaped interaction partner, the cohesin complex, are necessary for TAD formation. The role of cohesin in forming TADs involves loop extrusion, where DNA sequences are thought to be extruded through the ring until they encounter convergently orientated CTCF-bound sites at the boundaries (Sanborn et al., 2015; Fudenberg et al., 2016). The boundaries of TADs often interact due to the CTCF-cohesin complex, and function to locally reduce interactions with neighboring TADs. Thus enhancer-promoter interactions can occur within the TAD in which they reside but appear to be largely insulated or blocked from inappropriate interactions with sequences in neighboring TADs. Perturbation of TADs by large sequence variants overlapping boundaries by can cause dysregulated gene expression due to out-of-place (ectopic) interactions between an enhancer and promoter (Lupiáñez et al., 2015; Narendra et al., 2015; Franke et al., 2016; de Bruijn et al., 2020). Altering distances between enhancers and promoters can have dramatic effects on expression of newly proximal genes and the natural target of enhancers (Dillon et al., 1997). In contrast, deletion of individual CTCF sites or TAD boundaries does not greatly affect gene expression (Despang et al., 2019; Paliou et al., 2019; Williamson et al., 2019). Conditional ablation of CTCF or cohesin leads to loss of TAD structure throughout the genome, however gene expression is largely maintained. Detailed analyses show that interactions between genes in the vicinity of TAD boundaries, or whose interacting sites are located near TAD boundaries are largely lost (Thiecke et al., 2020). So too are interactions between other promoters and their long-range interacting sites which are CTCF/cohesin dependent.

#### 1.3.1 Enhancer-promoter interactions

Interestingly, enhancer-promoter contact does not always drive gene expression (Jin et al., 2013; Ghavi-Helm et al., 2014; Andrey et al., 2017). Analysis of enhancers associated with differentiation-induced genes identified two mechanistic types of enhancer-promoter contacts (Rubin et al., 2017). The first class, referred to as “gained” interactions increased in contact strength during differentiation in concert with enhancer acquisition of the H3K27ac activation mark. The second “stable” class was characterized by pre-established promoter contacts with H3K27ac-marked enhancers in undifferentiated cells. The stable class was associated with cohesin, whereas the gained class was not, implying distinct mechanisms of enhancer-promoter contact formation.

Thus enhancer-promoter interactions and gene expression are maintained by a mix of cohesin-dependent and cohesin-independent mechanisms. Furthermore, although cell differentiation is often characterized by dynamic rewiring of promoter-enhancer loops (Freire-Pritchett et al., 2017; Siersbæk et al., 2017), some specific interactions do not contemporaneously drive gene expression. Rather these may be enhancers in waiting for differentiation, or environmental signals to play a role in enhanced expression (Burren et al., 2017).

Mutations in enhancers and other regulatory sequence and genome rearrangements that disrupt enhancer-promoter interactions often underlie disease and developmental malformations (Lettice et al., 2003; Benko et al., 2009; Bhatia et al., 2013; Uslu et al., 2014; Lupiáñez et al., 2015; Franke et al., 2016). Naturally occurring variation such as single nucleotide polymorphisms (SNPs) in regulatory elements can also lead to variations in gene expression. This can ultimately increase disease susceptibility. Attempts to predict which regulatory elements act on which promoter(s) based solely on proximity in the linear genome sequence often leads to incorrect assignment. Understanding the 3D contacts between regulatory sequences is essential to understand the gene or genes affected by common sequence variation in health and disease.

## 2 In humans, genetic variation exists in health and in disease

Humans differ from each other by millions of DNA sequence variants. These variants, in combination with environmental triggers, can lead to phenotypic variation between individuals. This can range from variation in morphology (height, body mass index), behavioral traits (alcohol consumption, risk tolerance), predisposition to complex diseases (type 1 diabetes, rheumatoid arthritis) and response to diet or medication.

Genetic variants can be classified as sequence variants or structural variants, according to their composition. SNPs, the most common type of sequence variant, are substitutions of a single nucleotide at a specific position in the genome and occur every 100–300 bases. Less frequent sequence variants include insertions or deletions (indels) of segments of DNA of up to 1 kilobases (kb). On the other hand, structural variants involve larger segments of DNA (1 kb–5 Mb), and include duplications, inversions, translocations and larger indels. Genetic variants can be described as common (minor allele frequency [MAF] > 5%) and rare (MAF < 5%), depending on how often they appear in the population (Rahim et al., 2008).

Technological advances in genotyping and next generation sequencing have enabled all but the rarest human genetic variants to be catalogued. Large scale international efforts such as the HapMap Project (Frazer et al., 2007) and the 1000 Genomes Project (Auton et al., 2015) have densely genotyped thousands of individuals, providing a comprehensive resource on human genetic variation for a variety of ancestries, freely available in public databases. The NIH-sponsored SNP database (dbSNP) contains over 1,000 million distinct variants, as of build 155 released in June 2021 (Sherry et al., 2001).

Multiple studies assessing the heritability of complex traits have demonstrated that genetic factors are involved in 40%–70% of susceptibility to most common diseases. Therefore, characterizing this relationship between genetic variation and disease risk is an area of enormous interest. Understanding the genetic basis of disease will have a profound impact in the improvement of human health over the next decade, by helping to predict and prevent disease, to implement personalized medicine and to identify new drug targets (Zeggini et al., 2019).

Thanks to a phenomenon called linkage disequilibrium (LD), it is not necessary to directly assay all SNPs across the genome to test their associations with disease. Genetic variants can be inherited together in “blocks” or haplotypes. This means that the presence of a variant at a particular position on the genome can predict or “tag” the presence of other variants that are correlated, or in high LD, with it. International consortia have characterized genetic variants and the architecture of LD blocks across multiple populations (Lander et al., 2001; Frazer et al., 2007; Auton et al., 2015; McCarthy et al., 2016). This, together with technological advances and reduced genotyping costs, has enabled the systematic search for disease risk loci *via* high-throughput genome wide association studies (GWAS).

## 3 Genome wide association studies identify single nucleotide polymorphisms associated with a disease or trait

GWAS are very large studies that test for differences in the allele frequency of SNPs between individuals with different phenotypes, with the aim of finding associations between genotypes and phenotypes. GWAS require very large sample sizes to pinpoint genome wide associations, since each individual SNP may only contribute a small effect to the overall risk of disease. Microarrays are commonly used for genotyping in GWAS. However, microarrays tend to only capture common variation. Next generation sequencing can be used to genotype rare variants, but this method remains costly for genotyping the large number of individuals required for sufficiently powered case control association studies. After careful quality control, statistical tests are performed to detect significant associations between genotypes and phenotypes. Typically, logistic regression models test for associations if the phenotype is binary (i.e., presence or absence of disease), while linear regression models are used for continuous traits such as blood pressure or height (Uffelmann et al., 2021). Results must be validated in an independent replication cohort, and meta-analyses are typically conducted to increase sample size and statistical power (Corvin et al., 2010).

For the past 15 years, GWAS have made tremendous advances in identifying risk loci - blocks of correlated SNPs significantly associated with the trait of interest - for most common diseases (Visscher et al., 2017; Claussnitzer et al., 2020). The first GWAS, published in 2005 and studying age-related macular degeneration, demonstrated that hypothesis-free genetic association testing can reveal novel biological mechanisms for complex diseases (Klein et al., 2005). Two years later, the Wellcome Trust Case Control Consortium, 2007 set the standard for the subsequent flurry of successful GWAS studies, with the publication of the largest GWAS at that time, including seven diseases (2007). Since then, more than 5,600 GWAS have been published, identifying over 370,000 associations (Buniello et al., 2019), with samples sizes that now surpass 1 million individuals (Lee et al., 2018; Jansen et al., 2019). Such large GWAS are facilitated by the availability of large publicly available genetic datasets, such as the United Kingdom Biobank (Bycroft et al., 2018).

GWAS have revealed that, for most complex diseases, genetic predisposition arises from the combination of hundreds of mostly common variants with modest individual effect sizes. The next big challenge is to biologically interpret these genetic associations, to realize the potential of GWAS to elucidate mechanistic insights of disease etiology, facilitate personalized medicine and aid the development of novel drugs. There are examples of successful translation of GWAS findings into the clinic. For example, the identification of genetic association of variants in the IL-12/IL-23 pathway with Crohn’s disease (Wang et al., 2009) led to the use of drugs targeting this pathway to treat the disease (Moschen et al., 2019). However, several factors have so far limited the widespread use of GWAS findings for clinical benefit (Tam et al., 2019). As described above, LD facilitates the discovery of risk loci, but makes it difficult to pinpoint the causal variant or variants within a given locus. This is complicated by the fact that risk loci can harbor multiple, independent causal variants. In addition, most GWAS variants map to non-coding regions of the genome; approximately 5% of risk SNPs directly affect the coding sequence of a gene (Farh et al., 2015; French and Edwards, 2020). This makes the biological interpretation of GWAS variants challenging. Finally, although GWAS loci have traditionally been annotated to the nearest gene, many risk loci contain multiple genes in the vicinity or map at very large distances from coding genes, complicating the identification of the true causal genes.

Multiple studies have now shown that SNPs identified by GWAS are enriched in enhancers and other regulatory elements, that are active in disease-relevant tissues and cell types (Trynka et al., 2013; Farh et al., 2015; Cano-Gamez and Trynka, 2020). Regulatory elements are generally bound to transcription factors in open chromatin, with nearby marks of active transcription such as histone modifications. These elements likely use chromatin looping to come into contact with their target genes (Gasperini et al., 2020). Considerable efforts have been made to catalog regulatory elements across the human genome, by mapping these chromatin features using techniques such as ATAC-seq, ChIP-seq and Hi-C, among others (Bernstein et al., 2010; Dunham et al.,2012; Stunnenberg and Hirst, 2016). The recent EpiMap project illustrates how such functional genomics data can be used to predict disease-relevant tissues, causal SNPs and their target genes. Boix et al. used data from the main large scale consortia studies such as ENCODE, Roadmap and IHEC to compile 10,000 epigenomic maps across multiple cell types and tissues, which were used to identify 540 traits with 30,000 associated genetic loci (Boix et al., 2021).

## 4 Identifying causal variants, cell type and target gene

### 4.1 Fine mapping of disease-associated regions

A “typical” GWAS locus contains a number of highly correlated SNP variants associated with disease, situated either intronic or intergenic to gene coding regions. To fully utilize and translate GWAS, each disease-associated genetic region must be followed up by identifying the casual variant(s), the target gene(s) and the relevant cell type(s) in which the variants act.

Statistical fine mapping can refine the causal variants in a region. Information from SNPs genotyped *via* microarray is used to “impute” the association from other untyped SNPs. This is achieved using knowledge of the underlying relationship between SNPs from large numbers of sequenced genomes, currently over 85 million on the Michigan Imputation Server (Das et al., 2016). In this way the direct genotyping of a relatively limited number of ∼500,000 SNPs can be expanded to assess the association of tens of millions of variants. From here, these SNPs can be statistically fine mapped, using conditional analysis to determine whether the association signal from a locus is best explained with a single, or multiple independent genetic effects.

Bayesian statistical methods are now routinely used to assign a probability as to whether an associated SNP is causal. This provides the scaffold of a locus on which to add functional annotation. For example, a complicated locus may have multiple independent associated signals, each made up of many (>20) SNP variants with roughly equal probability of being causal. Alternatively, the more straightforward loci may well only have a single association, made up of one or two SNPs that are likely to be causal based on Bayesian probabilities. This describes the “credible SNP set” for a locus, the number of SNPs that make up 99% (or 95%) of the probability (Schaid et al., 2018).

This Bayesian framework incorporates knowledge of the biological nature of the SNPs into the probability models (Udler et al., 2019). For example, higher prior probabilities can be assigned to SNPs that reside in enhancer regions shown to be important in disease such as T cell enhancers in rheumatoid arthritis (Farh et al., 2015), or SNPs that change pivotal transcription factors in a cell type known to be important in disease. This will then inform the SNP(s) that are likely to be important in disease risk, for a given cell type.

### 4.2 Building the evidence case for causality

As previously described, regulatory regions can act on genes over long distances, often “skipping genes” and not necessarily regulating the closest gene in the linear view of a chromosome. It is therefore important, and non-trivial, to assign the disease-associated SNPs residing within enhancers to the genes they regulate. This can be achieved by combining evidence from a number of genomic technologies to build up evidence of the gene, and cellular context, in which the credible SNPs could act (Delaneau et al., 2019; Ding et al., 2020; Orozco, 2022).

Initially, the credible SNPs can be physically mapped to regions of the genome that are open and active in particular cell types. Open and active non-coding regions of the genome are potentially important in gene regulation. These regions are generally mapped *via* ATAC-seq for openness and ChIP-seq for activity. DNA is tightly wound around histones in the nucleus. In order to be active, the DNA is unwound locally from histones, and the region demarked with histones that are modified, methylated or acetylated, to maintain the open structure. The open DNA is more accessible, such that a transposase enzyme can insert adapters into these regions, so that they are preferentially represented in sequencing analysis (ATAC-seq) (Buenrostro et al., 2015). Histones can be modified to indicate promoters, enhancers, active enhancers or silencing regions. Antibodies that target specific histone modifications (such as H3K27ac for active chromatin, or H3K4me1 for enhancers) can determine these different regulatory genomic regions, by enriching sequencing libraries (ChIP-seq) (Ernst and Kellis, 2017). Using these molecular technologies, it is now possible to map the different chromatin states in a wide range of cell types (Gasperini et al., 2020).

These data can be generated in individual research labs, especially for specialist cell types, conditions or cells from patient samples. General cell type data are available in publicly available databases. Large projects such as the ENCODE (ENCODE Project Consortium, 2012) and Epigenomics Roadmap (Bernstein et al., 2010) document the state of many cell types. For example, the ENCODE-Roadmap Encyclopedia has generated ten “ground level” data sets, including ChIP-seq and ATAC-seq and Hi-C, on over 500 tissue and cell types (Moore et al., 2020). These data can be viewed in easily accessible websites such as IHEC (https://ihec-epigenomes.org/) (Stunnenberg and Hirst, 2016) or EPIMAP (https://epimap.fr/kremlin-bicetre) (Boix et al., 2021), where SNPs can be readily mapped to areas of activity in different cell types and states.

In this way it is possible to narrow down a range of SNPs from the credible set that are found within regions of activity in certain cell types. Of course, SNPs may exhibit their true causal nature in cell types and states outside these standard ones, for example only under certain stimulatory conditions, in a given chronicity or from a patient with active disease.

The statistical and functional annotation of disease-associated SNPs provides a strong hypothesis as to which of the credible SNPs are likely functional, and the cell type in which they are likely to be active. Overlaying evidence of transcription factor binding sites, from resources such as JASPAR (https://jaspar.genereg.net) (Fornes et al., 2020), could also indicate how the SNP would regulate gene transcription. For example, the destruction of a key transcription factor binding site could impact the regulation of gene expression under certain cellular conditions.

### 4.3 From single nucleotide polymorphism to target gene and cell type

Next the disease-implicated enhancer/SNP is assigned a likely target gene. Again, many lines of evidence can contribute. As previously described, TADs may well restrict the domain of enhancer/gene contact. Determining the gene expression in the relevant cell type, within the TAD where the implicated enhancer resides, will narrow down the likely target genes. This can be achieved using RNA-seq data from specific cells and exploring cell type-specific expression databases (e.g., the Human Cell Atlas; https://www.humancellatlas.org/and the Single Cell Expression Atlas; https://www.ebi.ac.uk/gxa/sc/home). Correlation between the activity of the enhancer (*via* quantitative ATAC-seq) and the activity of the gene (*via* quantitative RNA-seq or quantitative ATAC-seq of the gene region) can increase confidence as to the gene/enhancer relationship (Gate et al., 2018; Yang et al., 2020).

A stronger relationship between the enhancer SNP and gene expression can be found with expression quantitative trait locus (eQTL) analysis. Here the SNP variant (for example A/G), is correlated with gene expression, where one allele (e.g., A), is consistently associated with increased gene expression (Albert and Kruglyak, 2015). Such compelling evidence suggests that the variant does indeed influence gene expression, and directly links the SNP and gene. These relationships are best observed in the GTeX consortium data (GTEx, 2013) (https://gtexportal.org). Issues with eQTL exist. For example, it can take hundreds of samples to expose a robust relationship between SNP and gene, and these relationships can change, sometimes dramatically, based on cell type and stimulation (Fairfax et al., 2014). Often RNA-seq experiments are not performed on truly “homogeneous” cell types (e.g., T cells) which could mask the relationship between SNPs and gene in more refined studies (e.g., stimulated T-reg cells). The disease risk variant may also only be highly correlated to the true eQTL functional SNP, so appearing to change the expression, but not being the SNP responsible. In this case, “colocalization” statistical analyses are required to prove that the lead GWAS variant is also the lead eQTL variant (Hormozdiari et al., 2016).

Other QTL analyses can give insight into the function of a disease-associated SNP. For example, the risk allele may be correlated with enhancer activity (ATAC-QTL) (Gate et al., 2018), splice variants (splQTL) (Garrido-Martín et al., 2021), protein levels (pQTL) (Yao et al., 2018) or even histone modifications (hQTL) (McVicker et al., 2013), in specific cell types. As the databases are expanded with more, and different types of samples, these relationships will offer strong clues as to the likely function of variants that increase the risk of disease.

High resolution chromatin capture techniques, such as PCHi-C, can determine the physical links between the GWAS implicated enhancers and the genes they regulate (Martin et al., 2015; Javierre et al., 2016; González-Serna et al., 2022). Overlaying the ATAC-seq, ChIP-seq, RNA-seq, and QTL data with chromatin interaction data can provide more confidence as to the gene/enhancer relationship, in the identified cellular context. The advances in chromatin technology allow for base pair resolution of DNA interactions, from small numbers of starting cells (Downes et al., 2021). This is important in relating the function of enhancers in particular cell types, such as specific clinical subtypes from patients with active disease, or in remission. This has the power to directly link enhancers to their target genes, in different cellular contexts, and adds a compelling layer of evidence as to how GWAS variants act to increase the risk of disease.

Using this stepwise approach, researchers can refine a complicated structure of many potentially causal variants, to a limited number (statistical fine mapping, functional mapping) in a limited number of cell types (cell type-specific activity of enhancers) impacting a limited number of genes (eQTL, chromatin interactions). This provides a robust hypothesis as to how the variant increases risk of the disease. These hypotheses can be investigated using genetic engineering technologies such as CRISPR in 3D tissue cultures or mice, to provide a model for disease risk.

## 5 Chromatin conformation capture approaches

Experimental methods to interrogate the 3D folding of the human genome can be divided into microscopy-based and biochemical approaches. Although throughput is limited, advanced microscopy techniques can capture the temporal and spatial dynamics of 3D genome organization (Boettiger et al., 2016; Williamson et al., 2016; Bintu et al., 2018; Chen et al., 2018; Gu et al., 2018; Gabriele et al., 2022). Here, we focus on high resolution biochemical approaches to map long-range chromosomal contacts, including gene regulatory contacts between enhancers and their target gene promoters. The most commonly used approaches are variants of capturing chromosome conformation (3C) (Dekker et al., 2002) that rely on fixation and proximity ligation, although methods without ligation (Beagrie et al., 2017), without crosslinking (Brant et al., 2016) or without crosslinking and ligation (Redolfi et al., 2019) have also been established.

In 3C, cells are fixed with a short distance crosslinker such as formaldehyde to “freeze” the 3D genome folding in its native state. Subsequently, the chromatin is digested with a restriction enzyme, with the choice of the restriction enzyme determining the resolution of the resulting chromatin interaction maps (Su et al., 2021). Proximity ligation between the overhangs created by restriction digestion then creates hybrid molecules between chromatin regions that were in close proximity at the time of crosslinking. The frequency of these ligation events, revealed by massively parallel sequencing, is a readout for how often the corresponding chromosomal regions are in close spatial proximity in a specific cell type.

Chromosome conformation capture methods can be one-to-one (3C) (Dekker et al., 2002), one-to-all (4C) (Simonis et al., 2006; Zhao et al., 2006), many-to-many (5C) (Dostie et al., 2006) or all-to-all high-throughput (Hi-C) (Lieberman-Aiden et al., 2009). In Hi-C, the ligation step is preceded by integration of biotin at restriction fragment overhangs, enabling the isolation of biotin-marked ligation products. As a result, complex Hi-C libraries contain all the chromosomal interactions within a cell population. This has led to the discovery of key principles and building blocks of 3D chromatin organization, including TADs (Dixon et al., 2012; Nora et al., 2012), and A and B compartments in which active and repressed regions spatially segregate (Lieberman-Aiden et al., 2009). However, Hi-C libraries are enormously complex, with an estimated 10^11^ independent ligation products between ∼4 kb fragments of the mouse or human genomes (Belton et al., 2012). This prevents the reliable identification of promoter-enhancer contacts with statistical confidence, unless Hi-C libraries undergo ultra-deep sequencing (Rao et al., 2014; Bonev et al., 2017). This limitation has been addressed by methods have been developed to enrich 3C for specific subsets of interactions, such as Capture-C (Hughes et al., 2014; Davies et al., 2016; Chesi et al., 2019), or Hi-C libraries (Dryden et al., 2014; Schoenfelder et al., 2015a; Mifsud et al., 2015; Sahlén et al., 2015). Promoter-Capture Hi-C (PCHi-C) specifically enriches promoter-containing ligation products from Hi-C libraries, allowing capture of promoter interacting regions for all (>22 K) promoters across the mouse genome (Schoenfelder et al., 2015a; Sahlén et al., 2015; Wilson et al., 2016; Siersbæk et al., 2017; Comoglio et al., 2018; Novo et al., 2018; Schoenfelder et al., 2018) and the human genome (Mifsud et al., 2015; Javierre et al., 2016; Freire-Pritchett et al., 2017; Rubin et al., 2017; Chovanec et al., 2021). A direct comparison between Capture-C and Capture Hi-C revealed that Capture Hi-C generates two to three times more usable and informative reads (Su et al., 2021), due to the fact that Capture-C enriches for un-ligated fragments (Sahlén et al., 2015).

Variants of 3C approaches have replaced restriction enzymes with micrococcal nuclease (Micro-C) (Hsieh et al., 2015), yielding chromatin interaction maps with nucleosome level resolution. Micro-C libraries can be enriched for interactions with selected bait loci (Micro Capture-C or MCC) (Hua et al., 2021) or continuous genomic regions (Tiled-MCC) (Aljahani et al., 2022), closely mimicking previously developed approaches to enrich 3C or Hi-C libraries for specific interactions.

Further, several methods have been developed to target 3D chromatin interactions of accessible regions in the genome, including OCEAN-C (Li et al., 2018), HiCoP (Zhang et al., 2020), NicE-C (Luo et al., 2022), and HiCAR (Hi-C on accessible regulatory DNA) (Wei et al., 2022).

Alternative approaches have combined proximity ligation with chromatin immunoprecipitation (ChIP) to isolate chromosomal interactions involving specific proteins. In one approach, ChIP is performed, then biotinylation enables isolation of ligation products bound by the protein of interest (Fullwood et al., 2009). This approach is called Chromatin Interaction Analysis by Paired-End Tag sequencing (ChIA-PET). HiChIP, another protein-centric chromatin conformation capture method (Mumbach et al., 2016; Mumbach et al., 2017), combines marking of ligation junctions with biotin, ChIP, and construction of a transposase-mediated library. HiChIP requires a lower input of cells, and generates more informative reads compared to ChIA-PET (Mumbach et al., 2016). Similarly, proximity ligation assisted ChIP-seq (PLAC-seq) seems to outperform ChIA-PET by switching the order of proximity ligation and chromatin shearing steps (Fang et al., 2016).

Importantly, Capture Hi-C does not rely on ChIP (in contrast to ChIA-PET, HiChIP and PLAC-seq), and is therefore capable of interrogating chromosomal interactions irrespective of protein occupancy. This is key for comparing 3D chromatin interactomes in specific genetic backgrounds, for example wild-type to knockout. Indeed, this approach has uncovered a strong inter-chromosomal spatial interaction network between Polycomb-bound and -regulated genes in mouse embryonic stem cells (Schoenfelder et al., 2015b).

## 6 Bioinformatic tools to process and integrate genome wide association studies and 3D genomics data

**FIGURE 2:**
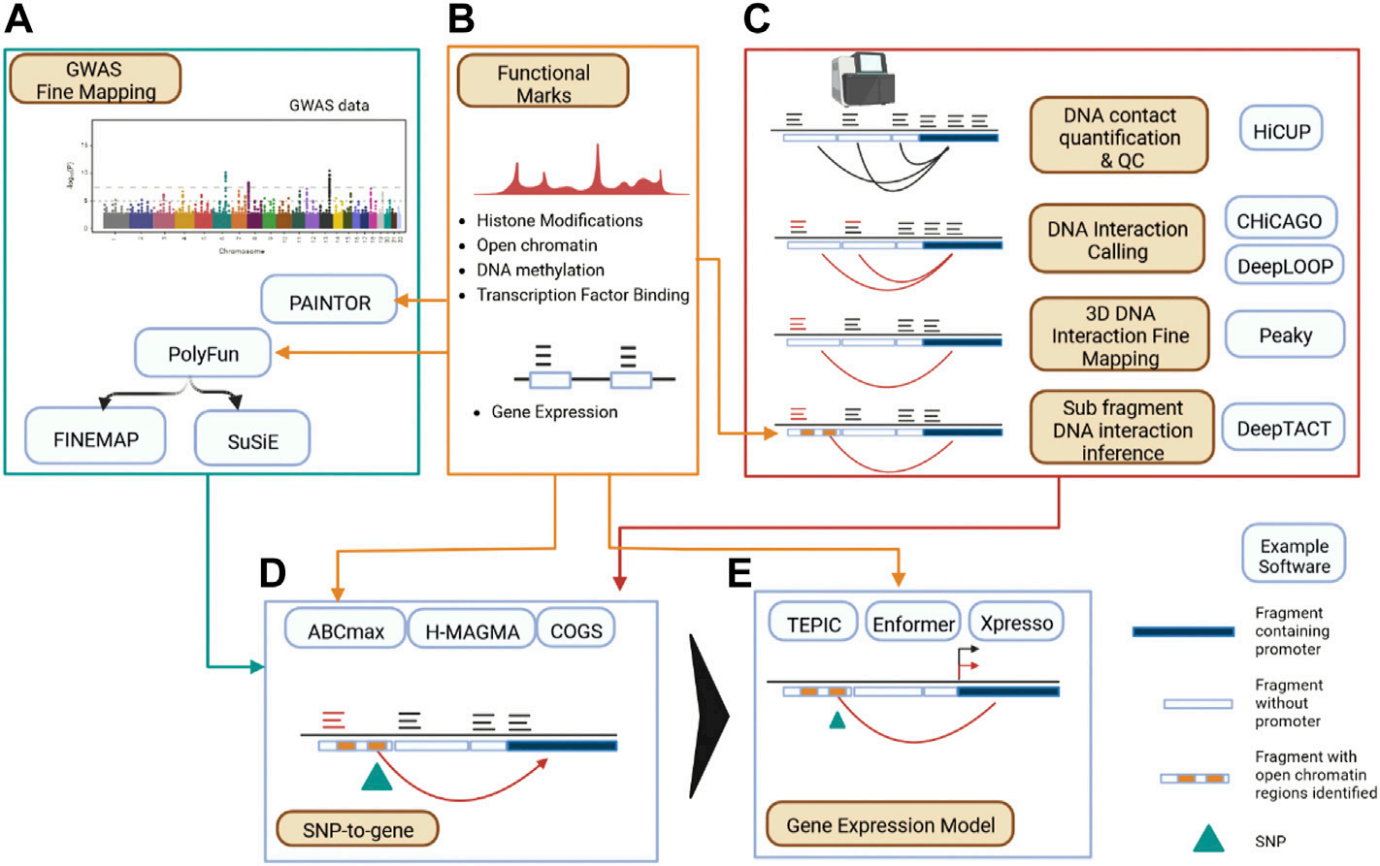
Data Processing Map for Applying 3D Functional Genomics to Disease SNPs. **(A)** GWAS summary statistics are fine mapped to form credible SNP sets, optionally employing functional information. **(B)** Functional datasets can inform multiple parts of a 3D genomics backed GWAS processing pipeline, informing GWAS fine mapping, DNA interaction calling, linking SNPs to genes, and modelling gene expression and regulatory networks. **(C)** Calling DNA interactions begins with alignment of sequencing reads to assign counts to linked restriction fragment pairs (light blue and dark blue partitions denote restriction fragments) giving a DNA contact profile (denoted by dark arcs). Functional DNA interaction calling can then be performed (denoted by red arcs). Further fine mapping of DNA interactions and inference of DNA interactions at the sub restriction fragment (denoted by yellow blocks) level is possible given additional functional data (such as ATAC-seq). **(D)** Linking disease associated SNPs (denoted by green arrow) to genes in a cell type specific manner. **(E)** Establishing the impact of genetic risk SNPs on gene expression.

Strategies to integrate GWAS and 3D genomics data as outlined in the previous sections have been supported by a maturing and expanded set of computational and statistical tooling over the last decade (Pal et al., 2019; Gong et al., 2021) (Table 1 ; Figure 2). These tools provide a base to analyze genetic disease risk in the context of the 3D functional genome; allowing insight into genetic contributions to disease (Gazal et al., 2022) and deep investigation into regulatory dynamics of complex diseases (Jin et al., 2013; Burren et al., 2017; Wang et al., 2018; Su et al., 2020). An analysis pipeline for mapping disease risk variants to target genes *via* 3D genomics seeks to establish confident *cis*-interactions, first by processing proximity ligation sequencing data to DNA contact data (Servant et al., 2015; Wingett et al., 2015) (Zheng et al., 2019), and then inferring functional relevance through the application of statistical models (Cairns et al., 2016; Mifsud et al., 2017; Ben Zouari et al., 2019; Holgersen et al., 2021). Several studies have evaluated the merits of common pipelines that implement these steps (Forcato et al., 2017; Aljogol et al., 2022), and one commonly used component (CHiCAGO) for calling biologically meaningful *cis*-interactions from DNA contact data generated from PCHi-C has been independently evaluated (Disney-Hogg et al., 2020) and guidance on usage published (Freire-Pritchett et al., 2021). Recent methodological advances allow for improved resolution over the typical restriction fragment level DNA interaction processing pipeline (Figure 2C). In one case, this has been achieved through development of a statistical model of neighboring DNA interactions so as to precisely assign DNA interactions to restriction fragments (Eijsbouts et al., 2019), another approach uses deep learning to infer sub-fragment *cis*-interactions using DNA sequence patterns and chromatin accessibility data (Li et al., 2019). Despite the challenges in calling *cis*-interactions from typically under-sampled Hi-C/CHi-C data (Aljogol et al., 2022), deep learning methods have been developed that provide improved DNA interaction coverage and resolution for sequencing and sample costs (Zhang, 2022).

**TABLE 1:** Selected software for integrating 3D functional genomes with disease SNPs.

Step	Software	
DNA contact processing	HiCUP	https://github.com/StevenWingett/HiCUP
	MHi-C	https://github.com/MHi-C
Promoter Interaction Calling	CHiCANE	https://cran.r-project.org/web/packages/chicane
	CHiCAGO	https://bitbucket.org/chicagoTeam/chicago
	CHiCMaxima	https://github.com/yousra291987/ChiCMaxima
	HiCapTools	https://github.com/sahlenlab/HiCapTools
	GOTHiC	http://www.bioconductor.org/packages/release/bioc/html/GOTHiC.html
Statistical and functional fine mapping	PolyFun	https://github.com/omerwe/polyfun
	SuSiE	https://github.com/stephenslab/susieR
	FINEMAP	http://www.christianbenner.com/
	PAINTOR	https://github.com/gkichaev/PAINTOR_V3.0
3D interaction fine mapping	Peaky	https://github.com/broadinstitute/ABC-Enhancer-Gene-Prediction
Gene Prioritization	ABC-max model	https://github.com/broadinstitute/ABC-Enhancer-Gene-Prediction
	COGS	https://github.com/ollyburren/rCOGS
	H-MAGMA	https://github.com/thewonlab/H-MAGMA
Gene expression prediction	TEPIC	https://github.com/SchulzLab/TEPIC
	Xpresso	https://github.com/vagarwal87/Xpresso
	Enformer	https://github.com/deepmind/deepmind-research/tree/master/enformer

Multiple reviews cover recent statistical advances that estimate causal genetic risk loci, accounting for LD and functional annotation (Spain and Barrett, 2015; Pasaniuc and Price, 2017; Hutchinson et al., 2020; Wang and Huang, 2022). Briefly, methods have evolved that use GWAS summary statistics rather than individual genotype level data, and relax the single causal variant per locus assumption of earlier landmark fine mapping work (Wakefield, 2009; Maller et al., 2012). Several of these methods allow for the integration of functional marks. For example, the posterior probability for causality computed by the fine mapping methods FINEMAP (Benner et al., 2016) and SuSiE (Wang et al., 2018) can be influenced using prior probabilities computed first using the PolyFun method (Weissbrod et al., 2020), while the PAINTOR method (Kichaev et al., 2014) is specifically designed to integrate functional annotation into the fine mapping process.

Having established credible SNP sets, *cis*-interactions can be used to link disease SNPs to target genes and estimate confidence of association with the disease (often referred to as a SNP-to-gene strategy—“S2G,” Figure 2D). One approach is to intersect fine mapped SNPs with Promoter Interacting Regions (PIRs) to establish a set of target genes. For example, Song et al (2019) first compute a window around each SNP such that neighboring SNPs must have LD coefficient of *r* ^2^ > 0.8, then intersects this window with PIRs to build a set of target genes (Song et al., 2019). Another strategy has been to develop a per-gene disease risk score using a framework that integrates all fine mapped genetic risk linked by PIRs (and other regions) to a particular locus, such as the COGS method (Javierre et al., 2016; Burren et al., 2017) and the more recent H-MAGMA framework (de Leeuw et al., 2015; Sey et al., 2020; Zhang, 2022). With the addition of functional datasets, the Activity By Contact (ABC) model for enhancer contribution to gene regulation (Fulco et al., 2019) can be employed to translate disease risk to the gene level. Credible SNP sets (for example, obtained from SuSiE) can be overlapped with ABC enhancers, and the gene with the highest ABC enhancer score is chosen (ABC-max) (Nasser et al., 2021). Combining multiple S2G strategies has been demonstrated to be important for biological insight (Giambartolomei et al., 2021) and accuracy (Gazal et al., 2022).

Finally, to estimate how a variant impacts the expressed genome, deep learning approaches that model gene expression from minimal input such as DNA sequence alone have recently undergone substantial improvement in prediction accuracy (Agarwal and Shendure, 2020; Avsec et al., 2021). However, representing long-range enhancer architecture appears to be critical for improving performance to a point where non-coding disease variants can be reliably assessed for their impact on RNA expression (Agarwal and Shendure, 2020). Avsec et al. (2021) also recently demonstrated that allowing for the representation of longer-range interactions in a DNA sequence based deep learning framework significantly improved gene expression prediction. Schmidt et al. (2020) demonstrate that the addition of inter-TAD promoter-enhancer contact data improves gene expression prediction using their existing TEPIC pipeline.

## 7 Future challenges

Although there has been substantial progress establishing technologies and bioinformatics tooling for 3D genomics over the last decade, multiple challenges and opportunities remain.

Recent evaluation of bioinformatics tooling demonstrates significant differences between tools used for calling *cis*-interactions from Hi-C (Forcato et al., 2017) and PCHi-C (Aljogol et al., 2022). In particular, these studies have highlighted low concordance between technical replicates and reproducibility trading off against resolution (e.g., window size of analysis) and the number of sample replicates. This can be attributed partly to under sampling of complex sequencing libraries from heterogenous cell populations. Technologies that allow for deconvolution of cell heterogeneity and increased efficient use of sequencing space will clearly empower all 3D genome applications. Bioinformatics methods to improve these characteristics are emerging, such as the aforementioned fine mapping of DNA interactions and machine learning derived models that will augment experimental measurement. Methods developed to predict *cis*-interactions from linear genome function marks will likely aid this effort (Piecyk et al., 2022). Thus, a core challenge for the field to enable diagnostic and therapeutic discovery applications is to drive cost-effective construction of 3D genome states at high resolution.

There are substantial improvements to gain in accurately linking SNPs to genes, as demonstrated by recent studies evaluating S2G strategies (Dey et al., 2022; Gazal et al., 2022; Lettre, 2022). These studies point to promoter-enhancer strategies (such as ABC models) along with wider profiling of multiple cell types/states as a promising avenue to pursue. Methodological improvements built on similar frameworks to ABC, combined with functional experimental paradigms may drive progress in this area. Thus, a further challenge in the field is the comprehensive integration of 3D genomes into SNP-to-gene linking strategies.

**FIGURE 3:**
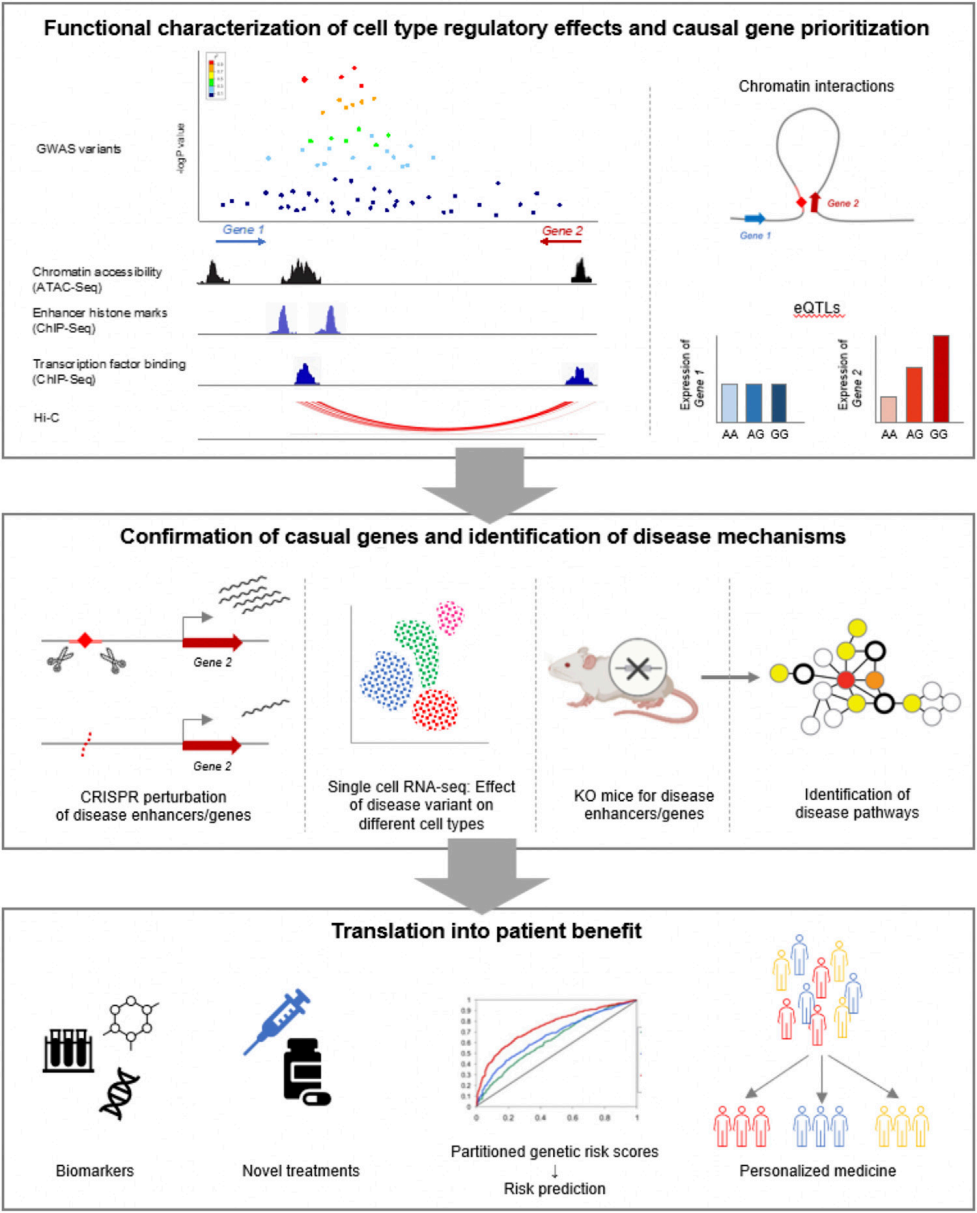
Overview of workflow for translating GWAS data into clinical benefit for patients.

3D genomes can also aid in inferring the functional impact of disease genetics on linked targets genes, such as the result on gene expression and effect across regulatory networks and pathways. The use of 3D genomics to provide data on long-range interactions (Agarwal and Shendure, 2020) as a base to construct transcriptional networks appears crucial to this goal. Thus, another challenge is the full integration of 3D genomes in the construction of transcriptional regulatory networks and pathways. Support for visualization and interactivity using 3D genomic data has advanced (Yardımcı and Noble, 2017; Oluwadare et al., 2019), but there is significant need for interactive systems focused on exploring disease risk genetics (Wlasnowolski et al., 2020), and to augment human designers of therapeutic and diagnostic strategies. These would allow for interactive exploration of scenarios based on genetic and structural configurations, enabling viewpoints at the base-pair level with the ability to simulate the impact on regulatory dynamics, pathways and cell state seamlessly. Finally, programs that support validation of disease genetics by profiling 3D genomes in multiple patient samples will be crucial to confirm and inform regulatory disease models established using 3D genomics (Figure 3).

Cleary, we are only just beginning to scratch the surface of the complete picture of how human genetic variation contributes to gene expression control, innumerable human traits and disease susceptibility across hundreds of different human tissue-types and their developmental and differentiation precursors. With continued improvement of molecular and computational technologies, and an ever-expanding fountain of new data we should expect to see significant new advances in our understanding of the causes and consequences of disease, which are the critical first steps in rational design of therapeutic interventions.

## Nomenclature

ABC: Activity by Contact
ATAC-seq: Assay for Transposase-Accessible Chromatin with high-throughput sequencing
ChIA-PET: Chromatin Interaction Analysis by Paired-End Tag sequencing
ChIP: Chromatin immunoprecipitation
ChIP-seq: Chromatin immunoprecipitation with massively parallel DNA sequencing
COGS: Common Omnibus Gene Score
CRISPR: Clustered regularly interspaced short palindromic repeats
eQTL: Expression quantitative trait locus
GWA: Genome wide association
GWAS: Genome wide association studies
Hi-C: High-throughput chromosome conformation capture
IBD: Inflammatory bowel disease
LD: Linkage disequilibrium
Micro-C/MCC: Micrococcal nuclease capturing chromosome conformation
PCHi-C: Promoter-Capture Hi-C
PIP: Posterior Inclusion Probability
PIR: Promoter interacting regions
PLAC-seq: Proximity ligation assisted ChIP-seq
QTL: Quantitative trait locus
RNA-seq: RNA sequencing
SNP: Single nucleotide polymorphism
TAD: Topologically associating domain
Tiled-MCC: Micro-Capture-C
